# Varietal variation and chromosome behaviour during meiosis in *Solanum tuberosum*

**DOI:** 10.1038/s41437-020-0328-6

**Published:** 2020-06-10

**Authors:** Anushree Choudhary, Liam Wright, Olga Ponce, Jing Chen, Ankush Prashar, Eugenio Sanchez-Moran, Zewei Luo, Lindsey Compton

**Affiliations:** 1 School of Biosciences, University of Birmingham, Birmingham, B15 2TT UK; 2 School of Natural and Environmental Sciences, Newcastle University, Newcastle upon Tyne, NE1 7RU UK; 3 Institute of Biostatistics, Fudan University, Shanghai, 200433 China

**Keywords:** Polyploidy in plants, Cytogenetics, Polyploidy in plants

## Abstract

Naturally occurring autopolyploid species, such as the autotetraploid potato *Solanum tuberosum*, face a variety of challenges during meiosis. These include proper pairing, recombination and correct segregation of multiple homologous chromosomes, which can form complex multivalent configurations at metaphase I, and in turn alter allelic segregation ratios through double reduction. Here, we present a reference map of meiotic stages in diploid and tetraploid *S. tuberosum* using fluorescence in situ hybridisation (FISH) to differentiate individual meiotic chromosomes 1 and 2. A diploid-like behaviour at metaphase I involving bivalent configurations was predominant in all three tetraploid varieties. The crossover frequency per bivalent was significantly reduced in the tetraploids compared with a diploid variety, which likely indicates meiotic adaptation to the autotetraploid state. Nevertheless, bivalents were accompanied by a substantial frequency of multivalents, which varied by variety and by chromosome (7–48%). We identified possible sites of synaptic partner switching, leading to multivalent formation, and found potential defects in the polymerisation and/or maintenance of the synaptonemal complex in tetraploids. These findings demonstrate the rise of *S. tuberosum* as a model for autotetraploid meiotic recombination research and highlight constraints on meiotic chromosome configurations and chiasma frequencies as an important feature of an evolved autotetraploid meiosis.

## Introduction

The global population is expected to increase from the current 6.7 to 9.8 billion by 2050, requiring a 56% food gap to be filled compared with the number of crop calories produced in 2010 (Searchinger et al. 2018). To meet this demand, a crucial goal is to develop crop-breeding strategies designed to effectively utilise the genetic variation that is currently locked up in crop plant genomes. The shuffling of genetic variation, the raw material for both natural and artificial selection, occurs during meiosis, a central process in the life cycle of sexually reproducing organisms. During this process, the pair of homologous chromosomes form physical connections (chiasmata), the cytological manifestation of genetic crossovers (CO) that allows a reciprocal exchange of genetic material between homologues (meiotic recombination). In plants with large genomes, including the major food crops potato, wheat and barley, the proximal regions (near centromeres) are typically suppressed for recombination, implying that a substantial percentage of genes may locate in regions of poor recombination and remain tightly linked (Kunzel and Waugh 2002; Akhunov et al. 2012; Marand et al. 2017). Some of those genes may affect agriculturally important traits, thus hindering the efficiency of breeding. Developing a detailed understanding of the mechanisms of crossing over will open up the potential for its manipulation, and is therefore a chief goal of modern plant breeding (Blary and Jenczewski 2019).

Comprehensive understanding of the factors controlling meiotic chromosome behaviour during meiosis has been developed in recent decades, particularly through use of the model diploid plant *Arabidopsis thaliana* (Osman et al. 2018). For example, each pair of homologous chromosomes has at least one CO, called the “obligatory CO” (Bomblies et al. 2016). Formation of COs requires the synaptonemal complex, a tripartite structure composed of two chromosome axes and a central element, which brings the homologous chromosomes into close proximity (Higgins et al. 2005). COs may be produced from two coexisting pathways. For class I COs, the presence of a CO at a given position on the chromosome reduces the probability of another CO occurring nearby, the classical phenomenon of interference (Sturtevant 1915). Meanwhile, class II COs are interference independent (Copenhaver et al. 2002). Comparatively less is known about how meiosis adapts to the polyploid state, though more recently, *Arabidopsis* species have provided an ideal model for studying the additional complexities in meiosis introduced by polyploidy (Lloyd and Bomblies 2016; Morgan et al. 2020).

Two broad groups of polyploids have traditionally been distinguished. Autopolyploids are created from genetically homogeneous genomes as a result of within-species genome duplication events, while allopolyploids originate from the union of two or more genetically distinct genomes. However, this distinction is an oversimplification of the complete spectrum of possibilities (Parisod et al. 2010). In diploid or allopolyploid species, the chromosomes usually pair, synapse and recombine as bivalents, leading to a disomic pattern of inheritance. However, in autopolyploids, chromosomes may have more than one possible pairing partner in the formation of bivalents. Pairing may occur randomly, leading to polysomic inheritance, or involves preferential pairing between particular chromosomes (Stift et al. 2008; Bourke et al. 2017; Kamiri et al. 2018). Furthermore, an individual chromosome may pair with one or more homologous chromosomes at the same time, leading to the formation of multivalents (Sybenga 1975; Lloyd and Bomblies 2016). Multivalent formation may lead to double reduction, in which identical alleles carried on the sister chromatids may enter the same gamete, which can occur with a frequency of up to 25% in autotetraploid species having four sets of homologous chromosomes (Luo et al. 2006). In practice, meiotic pairing behaviour in autopolyploids typically shows exclusive bivalent pairing or a mixture of bivalent and multivalent chromosome pairings (Bomblies et al. 2016; Lloyd and Bomblies 2016).

The particular mode of inheritance involving multivalent and/or bivalent chromosome pairing has been investigated in many autotetraploid species using a combination of molecular marker data and cytogenetic analyses (Soltis et al. 1993; Stift et al. 2008; Stift et al. 2010; Kamiri et al. 2018). In some autotetraploids including bird’s-foot trefoil (Fjellstrom et al. 2001), blueberry (Qu et al. 1998) and kiwi (Wu et al. 2014), chromosomes pair and recombine exclusively or almost exclusively as bivalents, with quadrivalent formation occurring rarely (<10%). In many other species, including *Arabidopsis arenosa* (Lloyd and Bomblies 2016), leek (Jones et al. 1996), alfalfa (Quiros 1982), gooseberryleaf alumroot (Wolf et al. 1989) and smooth hawksbeard (Vincent and Jones 1993), multivalent chromosome associations are frequently observed in prophase I, yet rarely persist into metaphase I, indicating that chiasma formation has not occurred. Instead, the chromosomes associate predominantly or exclusively as bivalents in metaphase I. This restriction in the type of chiasma configurations, together with a reduction in the total frequency of chiasmata, is a predominant feature in the evolution of many stable autotetraploid species (Bomblies et al. 2016).

Alternatively, an evolved autotetraploid state may feature a high rate of quadrivalent formation in metaphase I, indicating chiasma formation between more than two homologues (Bomblies et al. 2016). For example, in various species from purple yam (Abraham et al. 2013) to peavines (Khawaja et al. 1997) and various grasses (Koul et al. 1999; Deniz and Dogru 2006), quadrivalent formation at metaphase I reaches 20–47%. A more extreme example is cock’s foot, in which the chromosomes mostly form quadrivalents, and the chiasma frequency in evolved autotetraploids is higher than in the diploids or newly formed tetraploids (McCollum 1958). The changes in chiasma patterns characterising autotetraploid evolution are therefore a species-specific phenomenon.

While much progress has been made in understanding how meiosis adapts to the autopolyploid state in model species, including *A. arenosa* (Yant et al. 2013; Bomblies et al. 2016), much less has been done to address the process directly in important autopolyploid crops, including autotetraploid potato, leek and sugarcane. In particular, cultivated potato (*Solanum tuberosum*) is the third most important global food crop after rice and wheat, and has been recognised by the UNFAO as a globally important food of the future (Hussain 2016). Most cultivated potatoes are autotetraploid (2*n* = 4× = 48), though diploid, triploid, pentaploid and hexaploid germplasm also exists (Gavrilenko 2007). Historically, cytogenetic studies have been difficult in potato species due to a large number of small chromosomes and difficulties identifying individual chromosomes (Gavrilenko 2007). More recently, chromosome-specific cytogenetic markers have been developed in potato and other species in the form of molecular marker-tagged bacterial artificial chromosomes (Dong et al. 2000; Tang et al. 2009) and oligonucleotide probes (Han et al. 2015; Qu et al. 2017; Braz et al. 2018). These advancements enable the meiotic behaviour of individual chromosomes to be tracked. In this way, it has been shown that tetraploid *S. tuberosum* variety Katahdin has a very high frequency of multivalents in prophase I (65–78%), reducing to 21–42% by late diakinesis/metaphase I (He et al. 2018). However, a detailed treatment of the frequency and distribution of chiasma in tetraploid potato varieties is lacking.

It is well-known that genetic variation among varieties can lead to variation in chromosome-pairing behaviour and both global and local CO frequencies in various species (Esch et al. 2007). These include rose (Bourke et al. 2017), *Arabidopsis* (Sanchez-Moran et al. 2002; López et al. 2012), maize (Bauer et al. 2013) and potato (Swaminathan 1954), and also animals, including mice (Dumont et al. 2009). However, the extent of variation in chiasma frequency in modern commercial *S. tuberosum* germplasm is largely unknown.

The main aim of this study is to characterise the extent of variation in meiotic chromosome pairing behaviour as evident through metaphase I configurations and the subsequent CO frequency in different varieties of an autopolyploid crop, thus addressing how meiosis may adapt to the autopolyploid state. We used four modern varieties of the cultivated potato (*S. tuberosum*) to create a cytological reference map of the stages of meiosis in diploid and autotetraploid varieties. Specifically, we used fluorescence in situ hybridisation (FISH) to label the 45S and 5S rDNA sequences on chromosomes 1 and 2, enabling chromosomal configurations and hence chiasma frequencies to be distinguished. We have demonstrated variety-specific and chromosome-dependent variation in the frequency of bivalent versus multivalent meiotic chromosome associations, which underpins variation in CO frequency. We found a clear preference for bivalent pairing of chromosomes in autotetraploid varieties, accompanied by a reduction in the CO frequency per bivalent compared with the diploid variety. Finally, given the important role of the chromosome axis and synaptonemal complex in stabilising meiosis in autopolyploids (Hollister et al. 2012; Bomblies et al. 2016; Morgan et al. 2020), we immunolocalised two of the key axis proteins, ASY1 and ZYP1, and found that polymerisation and/or maintenance of the axis may be compromised in the tetraploid varieties.

## Materials and methods

### Plant materials

Three autotetraploid and one diploid variety of the cultivated potato *S. tuberosum* were used in the study. Sante, Cara and Maris Peer are autotetraploid varieties from the large tetraploid germplasm panel described in Sharma et al. (2018), while Scapa is a diploid variety (http://varieties.ahdb.org.uk/). The three tetraploids belong to different kinship groups identified by Sharma et al. (2018) based on 8 K single-nucleotide polymorphisms (Supplementary Table S1). Maris Peer and Cara are closely related, but more distantly related to Sante, as shown by phylogenetic analysis (Sharma et al. 2018). Five individual plants of each variety were grown in loam-based compost under controlled environmental conditions with a daytime temperature of around 22 °C and a night-time temperature of around 14 °C using a 16-h light cycle.

### Bud fixation

Individual anthers were dissected from the flower and measured using a calibrated graticule within the eyepiece of a dissecting microscope. Anthers were fixed in a 3:1 ratio of ethanol to glacial acetic acid fixative at 4 °C. The fixative was changed after 3–4 h and again after 24 h to remove chlorophyll (Armstrong et al. 2009). Meiotic stages in various anther sizes were assessed using anthers from five plants of each variety.

### Cytological analysis

Chromosomal spreads of pollen mother cells were prepared according to Armstrong et al. (2009), with a few minor modifications as follows. The fixed buds were washed with citrate buffer (pH 4.5) and incubated at 37 °C for 105 min in an enzymatic mixture containing 0.3% w/v pectolyase and 0.3% w/v cellulase in citrate buffer. After incubation, the enzyme was replaced by cold citrate buffer and a single anther was placed on a clean slide and macerated with a brass rod. About 10 μL of 80% acetic acid was added to the macerated anther and placed on the hot plate at 45 °C for 3 min while stirring with a needle and adding additional 80% acetic acid to prevent cells from drying. The slide was then removed from the hot plate, and cells were fixed with 200 μL of cold 3:1 ethanol to glacial acetic acid fixative and dried with a hair drier.

The cells were stained with DAPI (1 mg/mL) in Vectashield antifade mounting medium. FISH was carried out as per the protocol in Armstrong et al. (2009) using the 45S and 5S probes as described in Higgins et al. (2014). ASY1 and ZYP1 protein immunolocalisation was carried out using antibodies against *Arabidopsis* anti-rat AtASY1 and anti-rabbit AtZYP1 (kindly donated by FCH Franklin), as described in Armstrong et al. (2009).

### Chiasma scoring and statistical analysis

Cells in metaphase I were used to conservatively score chiasma on short and long chromosome arms based on the observed configurations and localisation of FISH signals, as described in Sanchez-Moran et al. (2001). The 5S rDNA repeats are present in the short arm of the submetacentric chromosome 1, proximal to the centromere. The 45S rDNA repeats are present at the distal end of the short arm of the subtelocentric chromosome 2, close to the nucleolus organiser region (Dong et al. 2000; Braz et al. 2018).

All configurations were scored conservatively, unless it was visually very clear that an extra chiasma was involved. A ring was considered to have two chiasmata and a rod to have one chiasma in diploids. In tetraploids, various configurations were possible. For example, a ring quadrivalent was considered to have four chiasmata and a chain quadrivalent to have three chiasmata.

Since chiasma data take the form of counts, with only a limited set of possible values, a non-parametric statistical test is required. We used the Kruskal–Wallis test to compare chiasma frequencies on each chromosome between potato varieties. Comparisons were made: (a) overall, which included all configurations (bivalent and multivalent) and was not normalised for the number of homologous chromosomes present; (b) on a per-bivalent basis, using those cells in tetraploids, which showed two bivalents for the chromosome of interest; (c) per chromosome, where the overall chiasma frequency was divided by four for tetraploid varieties or by two for the diploid variety. Post hoc pairwise comparisons were made using the Dunn test, applying a Bonferroni correction.

### Bioinformatic identification of potato ASY1 and ZYP1 proteins

*Arabidopsis* protein sequences were blasted in the Spud DB Potato Genomics Resource (http://solanaceae.plantbiology.msu.edu/cgi-bin/annotation_search.cgi) to identify and compare the potato-predicted protein sequences. The potato homologue of *A. thaliana* ASY1 (AT1G67370.1) was identified as Meiotic asynaptic mutant 1 (PGSC0003DMP400050690), and the potato homologue of *A. thaliana* ZYP1a (AT1G22275.1) and ZYP1b (AT1G22260.1) was identified as the ribosome-binding protein PGSC0003DMP400011376.

## Results

### Meiotic stages in diploid and tetraploid *S. tuberosum*

**Fig. 1:**
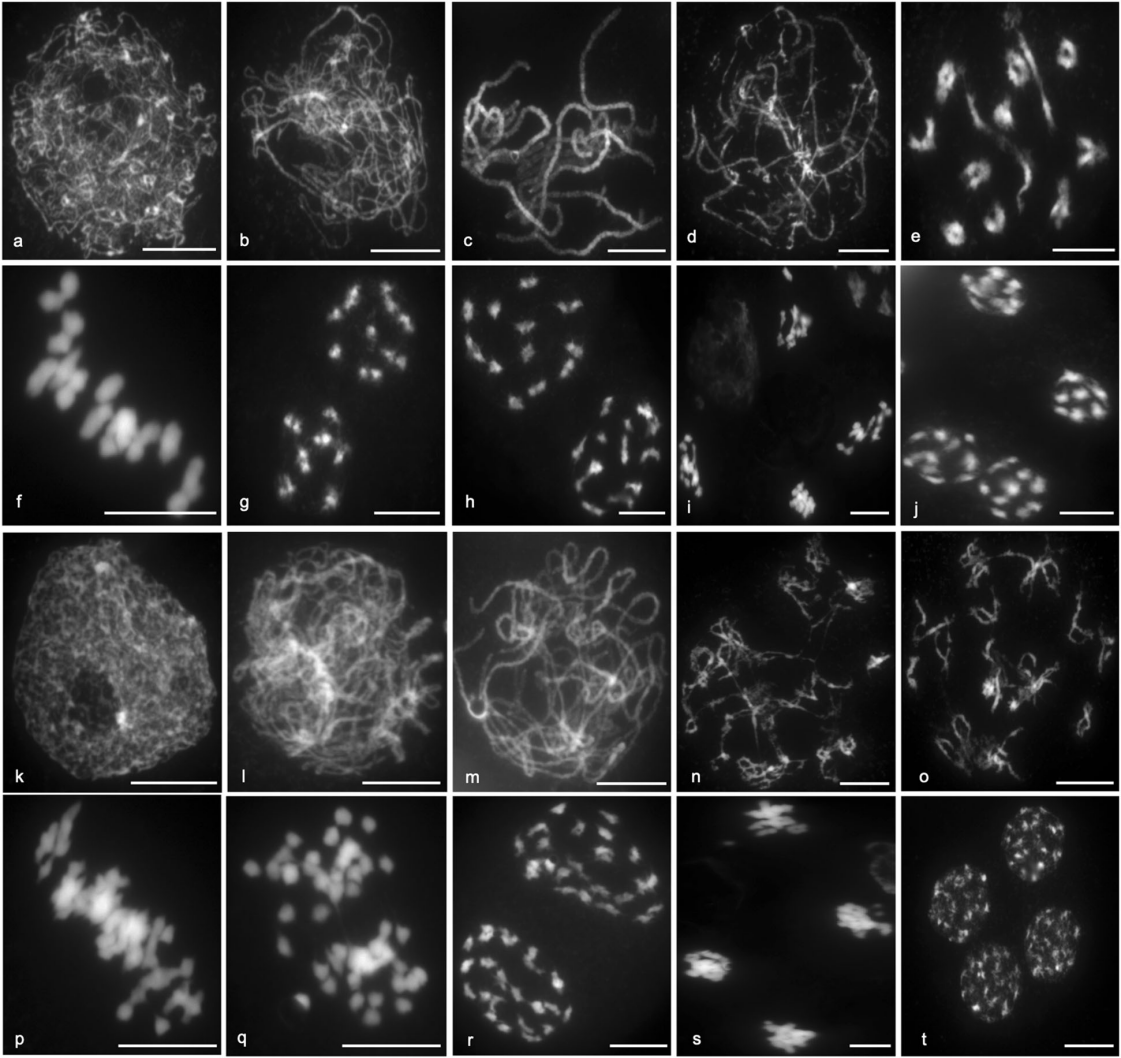
Stages of meiosis in diploid and tetraploid *Solanum tuberosum*. Meiotic stages are shown for Scapa (2×, **a**–**j**) and Maris Peer (4×, **k**–**t**), including leptotene (**a**, **k**), zygotene (**b**, **l**), pachytene (**c**), late zygotene/pachytene-like (**m**), diplotene (**d**, **n**), diakinesis (**e**, **o**), metaphase I (**f**, **p**), anaphase I (**g**, **q**), metaphase II (**h**, **r**), anaphase II (**i**, **s**) and telophase II or tetrad stage (**j, t**). Scale bars = 10 µm.

The stages of meiosis are shown for diploid Scapa (Fig. 1), and the three tetraploid varieties: Maris Peer (Fig. 1), Cara and Sante (Supplementary Fig. S1). At the metaphase I stage, twelve bivalents were usually clearly visible in diploid Scapa (Fig. 1f). In the tetraploids, 24 bivalents could be readily identified in some cells (Supplementary Fig. S1), though in other cells, a more crowded arrangement made it difficult to identify bivalents and/or quadrivalents (Fig. 1p).

Anther size was a reliable indicator of meiotic stage in all varieties, as shown in Supplementary Table S2. Meiosis was initiated (G2 to leptotene) in buds with similar size in both the diploid and tetraploid varieties. However, progression through prophase I into metaphase I may be delayed in the tetraploids, or there may be a loss of synchronisation of meiotic cells. For example, in tetraploid cells, metaphase I was still observed in buds larger than 2.0 mm, while in diploid cells, it was only observed in buds smaller than 2.0 mm (Supplementary Table S2). In addition, it was clear from the ease of isolating particular meiotic stages that cells spend a long time in zygotene, while pachytene is likely to be transient. Indeed, only late zygotene/pachytene-like cells could be observed in tetraploids (Fig. 1m and Supplementary Fig. S1), while a complete pachytene was observed in the diploid (Fig. 1c).

### Chromosomal configurations in diploid and tetraploid varieties

**Fig. 2:**
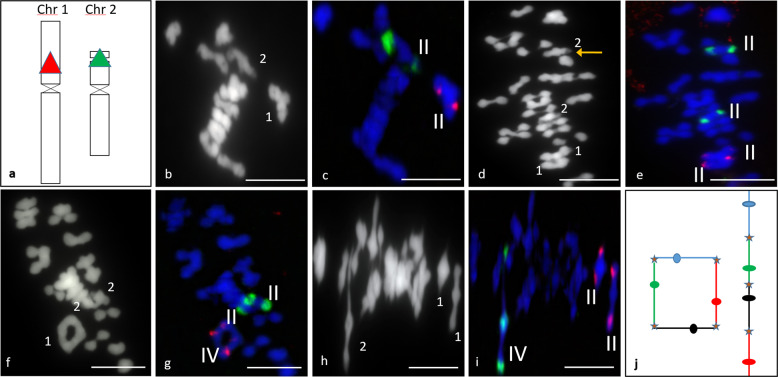
Bivalent and quadrivalent metaphase I chromosome configurations in diploid and tetraploid *Solanum tuberosum*. FISH signals were detected in metaphase I cells using 5S rDNA probe (red) for chromosome 1 and 45S rDNA probe (green) for chromosome 2 as shown in the chromosome ideogram (**a**). Bivalent pairing (II) can be seen in diploid Scapa (2×, **b**, **c**), and tetraploid Sante (4×, **d**, **e**). A yellow arrow in (**d**) indicates the decondensed rDNA region of chromosome 2. Quadrivalent pairing (IV) may be observed as a ring for chromosome 1 in tetraploid Sante (4×, **f**, **g**) or a chain for chromosome 2 in tetraploid Maris Peer (4×, **h**, **i**), both of which are illustrated in (**j**), where the four homologous chromosomes are shown in red, green, blue and black, and crossovers are marked with a star. Chromosomes have been stained with DAPI (blue). Scale bars = 10 µm.

We next conducted FISH analysis on chromosomal spreads in metaphase I using 5S and 45S rDNA probes, which localised the short arms of the submetacentric chromosome 1 and the subtelocentric chromosome 2, respectively, as shown in Fig. 2a. Chromosome 1 is the largest potato chromosome at ~90 Mb, while potato 2 is the smallest at ~50 Mb (Xu et al. 2011; Braz et al. 2018). By focusing on the location of the probe signals in metaphase I spreads, we could observe the chromosomal configurations involved in CO events and infer the minimum number of underlying chiasmata required to produce the observed configurations.

**Fig. 3:**
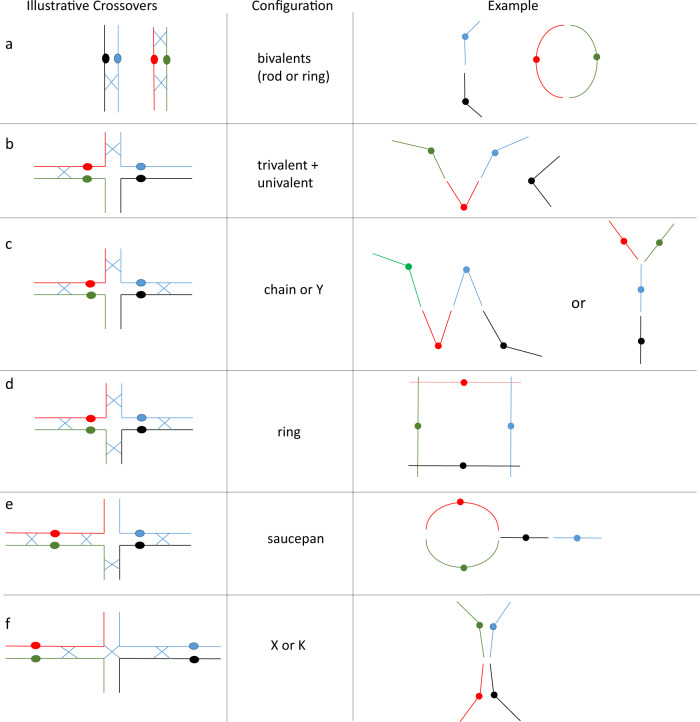
Illustrative chromosomal configurations in an autotetraploid meiosis. The four homologous chromosomes in an autotetraploid genome are shown in red, green, blue and black. The minimum number of required crossovers are marked with a blue X and solid circles depict centromeres.

In a diploid, the most likely configuration at metaphase I is a bivalent formed by the homologous chromosome pair. The bivalent may take two basic shapes depending on the number and location of chiasmata/COs. A rod bivalent results from at least one chiasma holding the homologues and leaving one of the arms free, while a ring bivalent results from at least two chiasmata involving both short and long chromosome arms (Fig. 3a) as also shown in Sanchez-Moran et al. (2001). Figure 2b, c shows a single-rod bivalent for each of chromosomes 1 and 2 in diploid Scapa (2x).

In contrast, in an autotetraploid, the presence of four homologues for each chromosome allows a wide spectrum of additional configurations involving CO formation between more than two homologues, leading to multivalent associations at metaphase I (Fig. 3b–f). For example, depending on the number and location of COs, the chromosomes may form a trivalent plus a univalent (two COs involving three chromosomes, Fig. 3b), a chain quadrivalent (three COs involving four chromosomes, Fig. 3c), a ring quadrivalent (four COs involving four chromosomes, Fig. 3d), a saucepan quadrivalent (four COs, Fig. 3e) or an X/K quadrivalent (three COs, Fig. 3f). Additional COs and other configurations may also be possible. Figure 2d, e shows two rod-shaped bivalents for each chromosome in tetraploid Sante (4×). The short arm of chromosome 2 is decondensed and stains faintly with DAPI, as observed previously (Braz et al. 2018) and indicated with a yellow arrow in Fig. 2d. In Fig. 2f, g, chromosome 1 formed a ring (illustrated in Fig. 2j), while chromosome 2 showed two rod bivalents. Figure 2h, i shows a chain quadrivalent formed by chromosome 2 in Maris Peer (illustrated in Fig. 2j), while chromosome 1 formed two bivalents (one rod and one ring). Quadrivalent formation was also observed for other chromosomes, which were not probed.

**Fig. 4:**
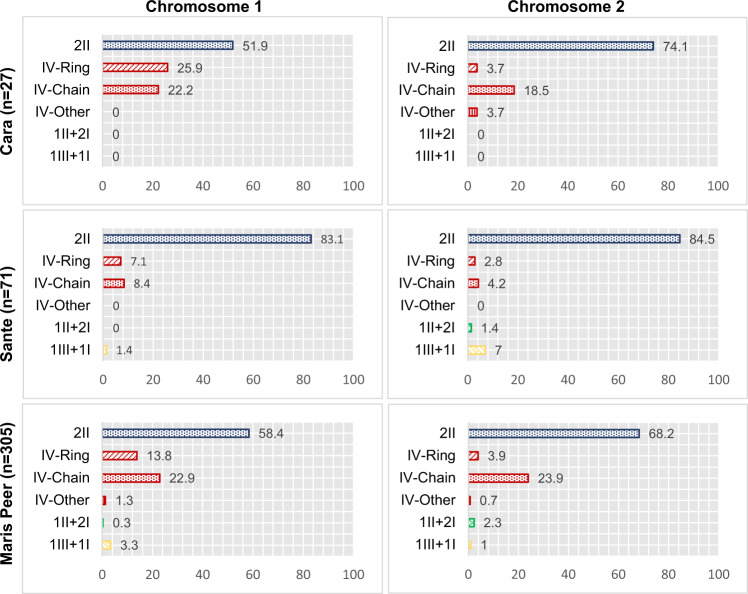
Chromosomal configurations at metaphase I in three tetraploid *Solanum tuberosum* varieties. I, II, III and IV indicate univalent, bivalent, trivalent and quadrivalent, respectively. The number of pollen mother cells analysed is given by *n*. The percentage of cells showing each configuration is indicated on the *x* axis.

We analysed the metaphase I configurations formed by chromosomes 1 and 2 in 403 pollen mother cells collected from five plants of each of the three tetraploid varieties (Fig. 4). For both chromosomes in all varieties, bivalent formation was more frequent than quadrivalent formation, deviating significantly from the expected 2:1 ratio of multivalents to bivalents (Supplementary Table S3) under a random-end model (Sybenga 1975). The frequency of quadrivalents varied widely from 7.0 to 48.1%, and was consistently higher for chromosome 1 than for the smaller chromosome 2. The frequency of quadrivalent formation was similar between Cara and Maris Peer, ranging from 38.0 to 48.1% for chromosome 1 and 25.9 to 28.5% for chromosome 2. However, variety Sante showed a significantly lower frequency of multivalents for chromosome 1 (15.5%) based on a 2-sample proportion test in comparison with either Maris Peer (*Z* = 3.84, *p* value = 0.0001) or Cara (*Z* = 3.17, *p* value = 0.002). Similarly, the multivalent frequency was significantly lower for variety Sante on chromosome 2 (7.0%) when compared with Maris Peer (*Z* = 2.65, *p* value = 0.0081). Overall, chain quadrivalents were more common than ring quadrivalents for both chromosome 1 (61.4% chains) and chromosome 2 (84.8% chains). Ring quadrivalents were significantly less frequent for chromosome 2 than for chromosome 1 (*Z* = 4.28, *p* value = 8.07 × 10^−5^). Examples of the rare chromosome 2 ring quadrivalents are shown in Supplementary Fig. S2. Configurations involving either a single univalent with a trivalent or two univalents with a bivalent were relatively rare in all three tetraploid varieties (Fig. 4).

### Chiasma analysis in diploid and tetraploid varieties

Based on the metaphase I configurations observed in 403 tetraploid pollen mother cells and 236 diploid pollen mother cells (Figs. 2 and 3), we carried out a chiasma count for chromosomes 1 and 2 (Table 1) using the conservative scoring method (see “Materials and methods”). The mean chiasma frequency was consistently higher for chromosome 1 than for chromosome 2 in all varieties. For both chromosomes, fewer chiasmata were always observed in the short arm compared with the long arm (Table 1).

**Table 1:** Mean chiasma frequency for chromosomes 1 and 2 in four *S. tuberosum* varieties.

Variety	Chiasma frequency	Chromosome 1 (4×/2×)				Chromosome 2 (4×/2×)			
		Short	Long	Total	*n*	Short	Long	Total	*n*
Cara (4×)	Overall	1.11 (2.2)	1.96 (2.0)	3.07 (2.1)	27	0.48 (2.7)	2.00 (2.1)	2.48 (2.2)	27
	Per bivalent	0.36 (0.7)	0.96 (1.0)	1.32 (0.9)	14	0.10 (0.6)	1.00 (1.0)	1.10 (1.0)	20
	Per multivalent	1.54	2.00	3.54	13	1.29	2.00	3.29	7
	Per chromosome	0.28 (1.1)	0.49 (1.0)	0.77 (1.0)	27	0.12 (1.3)	0.50 (1.0)	0.62 (1.1)	27
Sante (4×)	Overall	0.62 (1.2)	1.97 (2.0)	2.59 (1.8)	71	0.21 (1.2)	1.92 (2.0)	2.13 (1.9)	71
	Per bivalent	0.23 (0.5)	0.99 (1.0)	1.22 (0.8)	59	0.03 (0.1)	1.00 (1.0)	1.03 (0.9)	60
	Per multivalent	1.42	1.91	3.33	12	1.20	1.50	2.70	10
	Per chromosome	0.15 (0.6)	0.49 (1.0)	0.65 (0.9)	71	0.05 (0.6)	0.48 (1.0)	0.53 (0.9)	71
Maris Peer (4×)	Overall	0.94 (1.9)	1.92 (2.0)	2.87 (2.0)	305	0.40 (2.2)	1.93 (2.0)	2.32 (2.0)	305
	Per bivalent	0.32 (0.6)	0.97 (1.0)	1.29 (0.9)	178	0.04 (0.2)	0.97 (1.0)	1.02 (0.9)	208
	Per multivalent	1.35	1.93	3.28	126	1.15	1.98	3.11	90
	Per chromosome	0.24 (1.0)	0.48 (1.0)	0.72 (1.0)	305	0.10 (1.1)	0.48 (1.0)	0.58 (1.0)	305
Scapa (2×)	Overall (per bivalent)	0.50	0.97	1.47	236	0.18	0.97	1.15	236
	Per chromosome	0.25	0.49	0.74	236	0.09	0.49	0.57	236

The mean chiasma frequency is given for each variety on short and long chromosome arms and in total. In the diploid, the overall chiasma frequency is equivalent to the per-bivalent chiasma frequency because each cell has one bivalent. For tetraploid varieties, the overall chiasma frequency includes all pollen mother cells, the frequency per bivalent includes cells with two bivalents for the given chromosome and the frequency per multivalent includes cells with a multivalent for the given chromosome. The chiasma frequency per chromosome is obtained by dividing the overall frequency by four in tetraploids, or by two in the diploid. In brackets is the fold change between the corresponding tetraploid and diploid chiasma frequencies (4×/2×).

The overall chiasma frequency per cell varied significantly between the four varieties for both chromosome 1 (Kruskal–Wallis *χ*^2^_df=3_ = 336.47, *p* < 1.27 × 10^−72^) and chromosome 2 (Kruskal–Wallis *χ*^2^_df=3_ = 403.96, *p* < 3.07 × 10^−87^). Post hoc pairwise comparisons showed that diploid variety Scapa had a significantly lower overall chiasma frequency than all three tetraploid varieties (Supplementary Table S4a). Overall chiasma frequency in the long arms of tetraploid chromosomes was approximately doubled (Table 1) compared with the diploid variety Scapa. These observations are consistent with naive expectation, given that the tetraploid varieties have twice as many homologous chromosomes as the diploid. On a per-chromosome basis, the chiasma frequency in the long arm of chromosome 1 or 2 is therefore the same in both diploids and tetraploids (Table 1, Supplementary Table S4b).

**Fig. 5:**
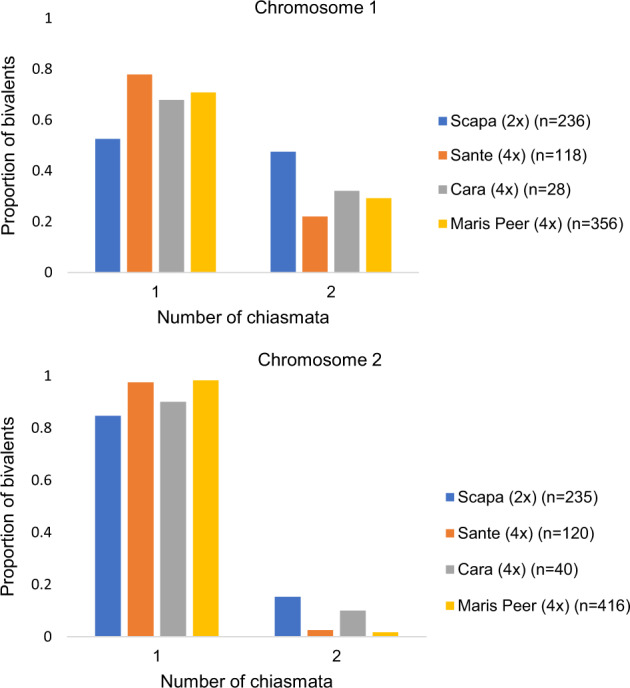
Variation in chiasma frequency per bivalent on chromosomes 1 and 2 among four varieties of *Solanum tuberosum*. The number of bivalents analysed is given by *n*.

Considering all tetraploid cells, with any bivalent or quadrivalent chromosomal configuration, variety Sante had a subtle (though non-significant) reduction in overall chiasma frequency on chromosomes 1 and 2 compared with the other two tetraploid varieties, Cara and Maris Peer. While the overall chiasma frequency was the same in the long arms of all three tetraploids, it was significantly reduced in Sante on the short arms of both chromosomes in comparison with Maris Peer, and for chromosome 1 in comparison with Cara (Table 1, Supplementary Table S4a). However, the chiasma frequency per bivalent was similar for all three tetraploids for both chromosomes 1 and 2 (Table 1, Fig. 5, Supplementary Table S4c), showing that variation in chiasma frequency between tetraploid varieties is largely underpinned by variation in the frequency of multivalent formation (Fig. 4).

By comparing tetraploid cells having two bivalents with diploid cells having one bivalent, we found significant variation in the per-bivalent chiasma frequency between the four varieties, for both chromosome 1 (Kruskal–Wallis *χ*^2^_df=3_ = 17.59, *p* < 5.34 × 10^−3^) and chromosome 2 (Kruskal–Wallis *χ*^2^_df=3_ = 23.31, *p* < 3.48 × 10^−5^). Post hoc pairwise comparisons showed that the per-bivalent chiasma frequency for chromosome 1 was reduced in all tetraploid varieties, and significantly so for Maris Peer and Sante, compared with the diploid variety Scapa (Table 1, Supplementary Table S4c). Likewise for chromosome 2, a reduction in the chiasma frequency per bivalent was observed for all three tetraploid varieties, though the difference was significant only for Maris Peer (*p* < 5.05 × 10^−5^).

Fig. 5 shows that two chiasmata per bivalent is a frequent event on chromosome 1 in diploid Scapa (47.5%), while in tetraploids, this frequency was reduced to only 27.8%. For the smaller chromosome 2, only 15.3% of bivalents had two chiasmata in Scapa, while this frequency reduced to only 4.7% in the tetraploids. For both chromosomes, the per-bivalent chiasma frequency on the long arm was equal to (or nearly equal to) one for all four varieties, accounting for the obligate CO in the majority of bivalents (Table 1). Meanwhile, Sante and Maris Peer were significantly less likely than the diploid to have an additional chiasma on the short arm, which explains the reduction in chiasma frequency per bivalent (Table 1, Supplementary Table S4c). The same pattern was also observed in Cara, though it was not statistically significant.

We also carried out a total chiasma count per cell for the limited number of pollen mother cells in which all 48 (in tetraploids) or 24 (in diploids) chromosomes could be observed as bivalent and/or quadrivalent associations (Supplementary Fig. S3, Supplementary Table S5a). The total chiasma frequency, including all 12 potato chromosomes, was approximately doubled in tetraploid variety Maris Peer compared with the diploid Scapa. In other words, the per-chromosome chiasma frequency (based on one copy of each chromosome) was approximately equal (Supplementary Table S5b). However, tetraploid Sante had a reduced overall chiasma frequency (27.4) compared with Maris Peer (30.3) (Supplementary Table S5a), echoing our findings from the FISH analyses of chromosomes 1 and 2. The per-chromosome chiasma frequency in Sante was significantly reduced compared with the diploid Scapa (Supplementary Table S5b). A similar reduction in chiasma frequency was also observed for chromosomes 1 and 2, due to fewer COs occurring on the short arms in Sante compared with the diploid (Table 1, Supplementary Table S4b).

### Immunolocalisation of the axis and synaptonemal complex proteins

ASY1 and ZYP1 are two important structural proteins, coordinating essential loading and interaction of various precursors and maintenance proteins to enable successful axis modulation and formation of the synaptonemal complex. In *Arabidopsis*, both ASY1 and ZYP1 are essential for normal chromosome synapsis, crossing over and proper segregation of chromosomes (Higgins et al. 2005; Sanchez-Moran et al. 2007). Defects in ZYP1, the transverse filament protein required for the formation of the synaptonemal complex, lead to a delay in the progression of meiosis and in meiotic recombination fidelity, leading to multivalent formation (Higgins et al. 2005). We identified the potato homologues of these proteins as meiotic asynaptic mutant 1 (PGSC0003DMP400050690), which showed 76% similarity to *Arabidopsis* ASY1, and PGSC0003DMP400011376, which is currently annotated as a ribosome-binding protein and showed 68% similarity to *Arabidopsis* ZYP1 (Supplementary Fig. S4).

**Fig. 6:**
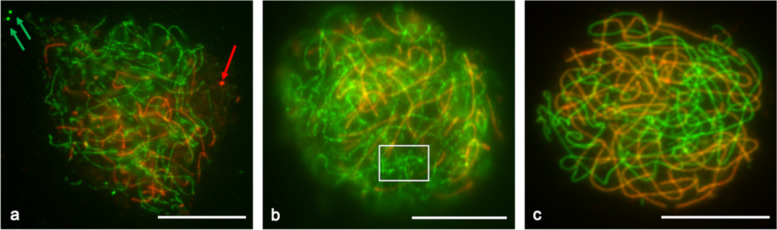
Immunolocalisation of meiotic axis protein ASY1 and synaptonemal complex central element protein ZYP1 in tetraploid *S. tuberosum* variety Sante and diploid variety Scapa. Late zygotene (pachytene-like) meiotic stages are shown for tetraploid Sante (**a**–**b**) and diploid Scapa (**c**). ASY1 is shown in green and ZYP1 in red. Red or green arrows in (**a**) point to large foci of the corresponding proteins, which are similar to polycomplexes described in other species. Chromosomal regions showing a patchy (not fully linear) distribution of ASY1 are highlighted in the white rectangle (**b**). Scale bars = 10 µm.

Immunolocalisation of ASY1 and ZYP1 was performed in potato using antibodies against the corresponding *Arabidopsis* proteins. While it was not possible to validate specificity of the antibodies due to unavailability of the necessary potato mutants, both ASY1 and ZYP1 signals localised to the chromatin in an expected manner. In both diploid and tetraploid varieties, the green ASY1 labelled the axis of the homologous chromosomes, while the red ZYP1 signal indicated formation of the SC central element (Fig. 6).

**Fig. 7:**
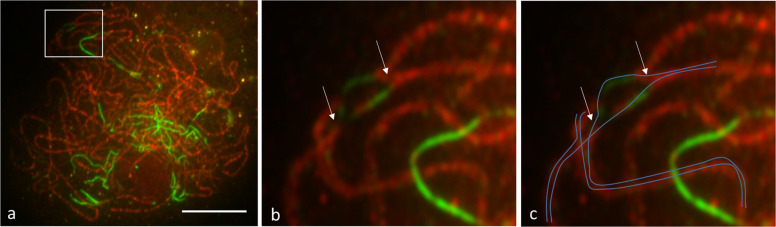
Late zygotene (pachytene-like) stage synaptic partner switching in tetraploid Maris Peer. ASY1 is shown in green and ZYP1 in red. The white square in (**a**) indicates the area enlarged in (**b**), where arrows indicate putative sites of synaptic partner switching. Blue lines in (**c**) depict an interpretation of the chromosomes undergoing partner switching. Scale bar = 10 µm.

In the immunolocalisation assays, we were not able to detect a full pachytene, as defined by a complete ZYP1 signal along all the chromosomes, in either diploid or tetraploid varieties, although pachytene cells could be found in the diploid (Fig. 1c). In the late zygotene (pachytene-like) tetraploid cells, we observed big foci of ASY1 and ZYP1 proteins, indicating a degree of structural difficulty in axis maintenance during prophase I (Fig. 6a) compared with the diploid (Fig. 6c). The ASY1 signal also appeared in patches of higher and lower intensity in the tetraploid (Fig. 6b) as compared with the diploid (Fig. 6c), i.e. not fully linear. In addition, sites of possible synaptic (pairing) partner switching were observed between the homologues in tetraploid potato, in which a chromosome may synapse with more than one partner simultaneously, as indicated by arrows (Fig. 7b, c).

## Discussion

### Variation in meiotic chromosome configurations in three autotetraploid *S. tuberosum* varieties

We developed a FISH method to analyse meiotic chromosome configurations and CO frequency for chromosomes 1 and 2 in three tetraploid varieties of *S. tuberosum*. A recent survey of 20 naturally evolved autotetraploid species (Bomblies et al. 2016) showed that there are three basic classes of autotetraploids: (1) most commonly, species that form exclusively or almost exclusively bivalents at metaphase I; (2) species that show a mixture of bivalents with quadrivalents; (3) most rarely, species in which quadrivalents predominate. We found that the three varieties of tetraploid potato belong to the bivalent-plus-quadrivalent class, with quadrivalent frequency varying from 7.0 to 48.1% across varieties and chromosomes, which is broadly similar to the frequencies observed previously on chromosomes 2, 4, 7 and 11 (21.3–42.1%) in variety Katahdin (He et al. 2018), as well as to estimates from molecular marker data (Bourke et al. 2015). It is interesting to note that the frequency of quadrivalent formation observed for chromosome 2 in variety Katahdin (He et al. 2018) is much higher (42%) than the frequencies we observed across all three varieties (7.0–28.5%), particularly given the small size of chromosome 2 and the use of Katahdin in breeding varieties Sante and Maris Peer. This difference could be due to a combination of genetic and environmental factors (Bomblies et al. 2015).

Quadrivalent formation was significantly less frequent in variety Sante, particularly on chromosome 1 (15.5%), compared with either Maris Peer (36.7%) or Cara (48.1%). These differences led to a lower overall CO frequency in Sante compared with the other two tetraploids, particularly on the short arms of both chromosomes. The overall CO frequency we observed on chromosomes 1 and 2 is similar to the frequencies estimated across all chromosomes in commercial diploid and tetraploid potato varieties (Magoon et al. 1962; Sangowawa 1989). Given that Cara and Maris Peer are more closely related to each other than to Sante (Sharma et al. 2018), the variation we observed in multivalent formation and CO frequency may be under genetic control, perhaps involving single-nucleotide polymorphism and/or copy number variation among varieties (Iovene et al. 2013; Sharma et al. 2018). In addition, the varietal variation may be related to different epigenomic features that have been associated with COs in potato, including open chromatin, H3K4me3 nucleosome modification and the distribution of Stowaway transposons (Marand et al. 2017).

In our study, and in plants in general (e.g. Lopez et al. 2012), the CO frequency is higher on longer chromosomes than on shorter chromosomes. For example, we observed an average chiasma frequency in three tetraploid varieties of 2.83 for the larger chromosome 1 (~90 Mb), compared with 2.30 for the smaller chromosome 2 (~50 Mb). From a diploid segregating population, the genetic map length for chromosome 1 (93.0 cM) is larger than that of chromosome 2 (77.4 cM). However, chromosome 1 is a similar length (36.7 Mb) to chromosome 2 (33.5 Mb) if the estimated length of pericentric heterochromatin is excluded (Sharma et al. 2013; Sharma et al. 2018). Further work addressing the chromatin state and distribution (proximal–distal) of COs along the chromosome arms in potato is warranted to address this observation. CO frequency was also higher on the long chromosome arms compared with the short arms. The presence of COs on the short arm of chromosome 2 is particularly noteworthy, given that chromosome 2 is subtelocentric, with a highly heterochromatic nucleolus organiser region and satellite regions in the small arm (Dong et al. 2000; Braz et al. 2018). Excluding the rDNA repeats within the nucleolus organiser region, chromosome 2 has an arm ratio of 3.63 ± 0.61, compared with ratios between 1.19 and 2.67 for the other eleven potato chromosomes (Braz et al. 2018), indicating the particularly small size of the short arm.

In the closely related species *S. lycopersicum* (tomato), the short arm of chromosome 2 also carries the nucleolus organiser region and is entirely heterochromatic (The Tomato Genome Consortium 2012). The arm ratio is also high (3.31 ± 1.37) compared with other tomato chromosomes (Braz et al. 2018). Many studies have established that there is no crossing over on the short arm of tomato chromosome 2 (Brown 1949; Barton 1951; Sherman and Stack 1995), which is consistent with reports in various organisms of a lack of crossing over in heterochromatin and nucleolus organiser regions (Sherman and Stack 1995, and references therein). However, a more recent study has shown evidence for class II but not class I recombination nodules on the short arm of tomato chromosome 2 (Anderson et al. 2014). Furthermore, in *A. thaliana*, the nucleolus organiser regions on the short arms of chromosomes 2 and 4 do form COs, which are likely to be enabled by stretches of euchromatin (Sanchez-Moran et al. 2001). Similar euchromatic regions could exist on potato chromosome 2, though the structure of the chromosome has not been analysed in detail here. We could further speculate that the COs we observe on the short arm of chromosome 2 may be class II COs, given that this type of CO is enriched within pericentromeric heterochromatin and short chromosome arms in tomato (Anderson et al. 2014). This question could be addressed in future work by developing further immunocytological methods to specifically mark class I and class II COs in potato.

We found that quadrivalent formation occurred more frequently for the larger chromosome 1 in all three tetraploids. Moreover, quadrivalents involving at least four COs (rings) were significantly less frequent than those requiring at least three COs (chains), particularly on chromosome 2. These observations are consistent with those in a range of other newly synthesised and natural polyploids, including *A. thaliana* (Santos et al. 2003), *Zea perennis* (Shaver 1962) and bird’s-foot trefoil (Dawson 1941; Davies et al. 1990). They also indicate the existence of strong interference along potato chromosomes, which has been shown previously (Park et al. 2007), and support a model in which an “interference signal” spreads out in either direction from designated CO sites, gradually decreasing in strength (Bomblies et al. 2016). Note that with the conservative chiasma-counting approach we used, there will inevitably be a degree of underestimation of CO frequency. However, we consider it unlikely in most cases that the observed configurations contained more than the minimum number of COs, given the above-described evidence for interference in potato.

### Comparison of diploid and autotetraploid chiasma frequency

It is well-known that the evolution of an autotetraploid species into a stable form often involves a reduction in the overall frequency of chiasmata compared with the diploid progenitors or with newly synthesised autotetraploids (Bomblies et al. 2016). Although the diploid variety Scapa used in this study is not the progenitor of the tetraploid varieties, it is nonetheless of interest to compare the diploid with the tetraploids. The number of pollen mother cells we analysed varied widely among varieties due to the difficulty in isolating metaphase I stage cells in both diploid and tetraploid varieties. It was clear from the relative ease of isolating prophase I stages that homologous identification, pairing and chiasma formation likely occupies the majority of time spent in meiosis in both diploids and tetraploids. In addition, by correlating anther size with observed meiotic stages, we saw evidence of delays in chromosome pairing and synapsis in tetraploid varieties compared with the diploid. However, the above observations require confirmation through a time-course analysis of meiosis, for example by applying a BrdU (5-bromo-2′-deoxyuridine) or EdU (5-ethynyl-2′-deoxyuridine) method (Stronghill et al. 2014).

Interpretation of the number of chiasmata from chromosomal configurations is challenging in diploid and tetraploid varieties alike. Although there is a broad spectrum of possible configurations in tetraploids, the number of chiasmata may be very clear, for example, when the four homologues form a ring multivalent, while it can be more difficult to decipher the number of chiasmata for ring bivalents in either diploids or tetraploids. Nevertheless, we have observed that CO frequency on chromosomes 1 and 2 (and per cell) in the diploid variety was approximately half that observed in the tetraploid varieties, which is similar to previous observations in the model plant *A. thaliana* (Parra-Nunez et al. 2019). The per-bivalent chiasma frequency was very similar for all three tetraploid varieties, varying from 1.22 to 1.32 for chromosome 1 and from 1.02 to 1.10 for chromosome 2. This is consistent with observations in other tetraploid species showing that the CO frequency per bivalent is reduced towards the minimum value of 1. For example, in *A. arenosa*, bird’s-foot trefoil and roundtip twinpod, the average frequency of chiasmata is 1.1 per bivalent (Mulligan 1967; Davies et al. 1990; Yant et al. 2013; Bomblies et al. 2016). Furthermore, the CO frequency per bivalent in tetraploids is lower than in diploid Scapa, due to a reduction in COs occurring on the short chromosome arms. While two COs per bivalent occurred frequently in the diploid, it was a relatively rare event in all three tetraploid varieties. These observations may support the hypothesis that an increase in “crossover interference distance” is a key feature in the evolution of chiasma patterns in autopolyploids (Bomblies et al. 2016). At the same time, we speculate that as a largely clonally propagated species, *S. tuberosum* may not be under the same evolutionary pressure as other wild autopolyploid populations to evolve a more stable, bivalent-dominated meiosis. This could explain why it retains a substantial proportion of multivalent chromosome associations.

### A potential disturbance of the chromosome axis in autotetraploid potato

Autotetraploid species may face various challenges during prophase I of meiosis as a result of all four homologous chromosomes being able to pair and synapse with each other, including entanglement or chromosome interlocks (Bomblies et al. 2016). Here, we have observed the absence of a complete pachytene stage in tetraploid potato, indicating incomplete synapsis and supporting previous observations in other species, including the wild tetraploid *Solanum hjertingii* (Sangowawa 1989) and leek (Khazanehdari et al. 1995).

The presence of four homologues means that synapsis can start to occur between two homologues, and then individual homologues can switch over to pair with other homologues, explaining why a complete synaptonemal complex between two homologues may not be observed in the immunolocalisation analyses. We observed such pairing partner switches in the late zygotene/pachytene-like stage in tetraploid potato varieties. These switches could be confirmed and visualised in greater detail using structured illumination microscopy, which has been applied in the established autotetraploid, *A. arenosa* (Morgan et al. 2020). In addition, we have observed large foci of ASY1 and ZYP1 proteins, which are similar to those described previously in yeast as polycomplexes (Sym and Roeder 1995; Henderson and Keeney 2004), and also observed in hexaploid wheat *ph1b* mutants (Boden et al. 2009), and in *A. arenosa* grown at high temperature (Morgan et al. 2017). This could indicate difficulties in axis polymerisation and/or maintenance in tetraploids. Polycomplexes could be formed after dissolution of the synaptonemal complex, or even before its formation, serving as a storehouse of the required proteins (Sym and Roeder 1995). It has been suggested that the central filament proteins can self-assemble when proper assembly and polymerisation of the synaptonemal complex are disturbed (De Carvalho and Colaiácovo 2006), for example due to overproduction of axis-related proteins. Further work is warranted to investigate axis dynamics in tetraploids, including investigation of whether the tetraploids ever show complete synapsis (i.e. a complete pachytene stage), and to compare the level of synapsis between diploids and tetraploids.

## Conclusions and future research

We conclude that meiosis in *S. tuberosum* is partially diploidised, with bivalent chromosome associations dominating at metaphase I. Reduction in the CO frequency per bivalent is likely to be an important feature driving the stabilisation of autotetraploid meiosis in potato. However, further work is required to look at all 12 potato chromosomes and to focus on anaphase I and II, to characterise the accuracy of chromosome segregation and subsequent pollen fertility. Given the evidence for strong interference in potato, it would be interesting to further characterise the role of the meiotic chromosome axis and synaptonemal complex in stabilising autotetraploid meiosis. Measurements of chromosome axis length could be employed to further test the hypothesis that an increase in CO interference distance in tetraploids is mediated directly or indirectly by changes in chromosome axis length (Bomblies et al. 2016).

Multivalent chromosome associations were also observed at metaphase I, at a considerable and variety-specific frequency. These findings imply that methods for the genetic analysis of complex traits (e.g. quantitative trait locus analysis) in autotetraploid species such as potato should be designed to accommodate crossing over in multivalents, and the consequent features of allele segregation, including double reduction (Chen et al. 2018; Chen et al. 2020). Finally, meiosis in autopolyploids has to adapt to the external as well as the internal environment (Bomblies et al. 2015); therefore, future work may focus on evaluating the generality of our findings not only in a larger more diverse potato germplasm collection, but also in field versus controlled growth environments.

## Supplementary information

Choudharyetal(2020)SupplementaryHDY-19-A0401RR

## Data Availability

Data available from the Dryad Digital Repository: 10.5061/dryad.59zw3r24n.
