# Supplementary material for: Varietal variation and chromosome behaviour during meiosis in Solanum tuberosum
Source: Heredity (Edinb). 2020 Jun 10;125(4):212–26. doi: 10.1038/s41437-020-0328-6 (PMC7490355; doi:10.1038/s41437-020-0328-6)
Supplement: Supplementary file 1 — Choudharyetal(2020)SupplementaryHDY-19-A0401RR [file 41437_2020_328_MOESM1_ESM.docx]

**Supplementary Material for Heredity Original Research Article:**

**Choudhary *et al*. (2020) Varietal Variation and Chromosome Behaviour during Meiosis in *Solanum tuberosum***

Anushree Choudhary^1^, Liam Wright^1^, Olga Ponce^1^, Jing Chen^1^, Ankush Prashar^2^, Eugenio Sanchez-Moran^1^, Zewei Luo^1,3^ and Lindsey Compton^1*^

^1.^ School of Biosciences, University of Birmingham, Birmingham B15 2TT, UK

^2.^ School of Natural and Environmental Sciences, Newcastle University, Newcastle upon Tyne, NE1 7RU, UK

^3.^ Institute of Biostatistics, Fudan University, Shanghai 200433, China

*Corresponding author

Dr Lindsey Compton

School of Biosciences

University of Birmingham

Edgbaston, Birmingham

B15 2TT, United Kingdom

Tel: +44 121 414 5883

E-mail: [l.j.compton@bham.ac.uk](mailto:l.j.compton@bham.ac.uk)

**Supplementary Figures**

**Fig. S1** Stages of meiosis in tetraploid *S. tuberosum* varieties Cara and Sante


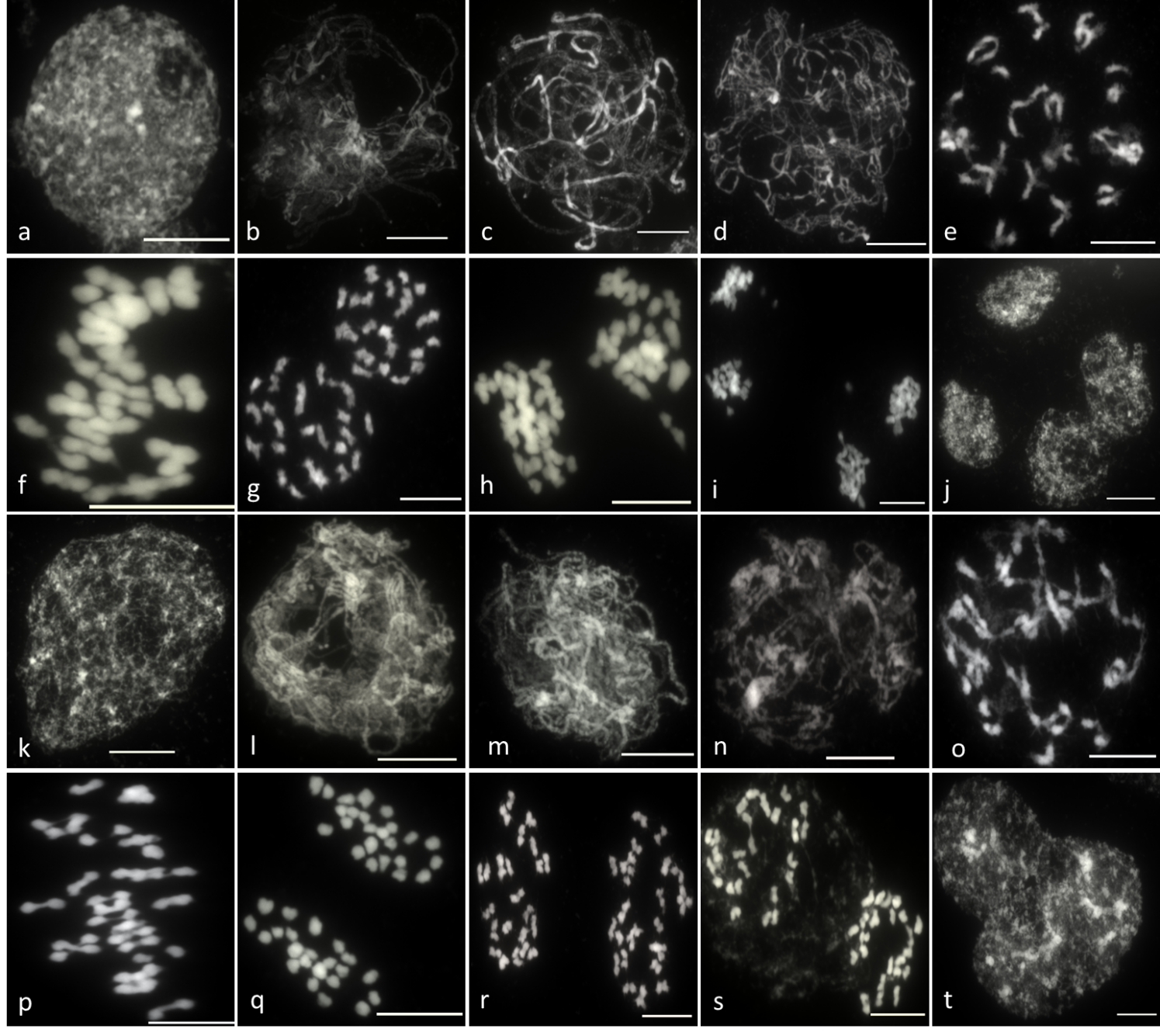


Meiotic stages are shown for Cara (**a-j**) and Sante (**k-t**) including leptotene (**a, k**), zygotene (**b, l**), late zygotene/pachytene-like (**c, m**), diplotene (**d, n**), diakinesis (**e, o**), metaphase I (**f, p**), anaphase I (**g, q**), metaphase II (**h, r**), anaphase II (**i, s**) and tetrad (**j, t**). Scale bars = 10µm.

**Fig. S2** Ring quadrivalent formation in tetraploid variety Sante


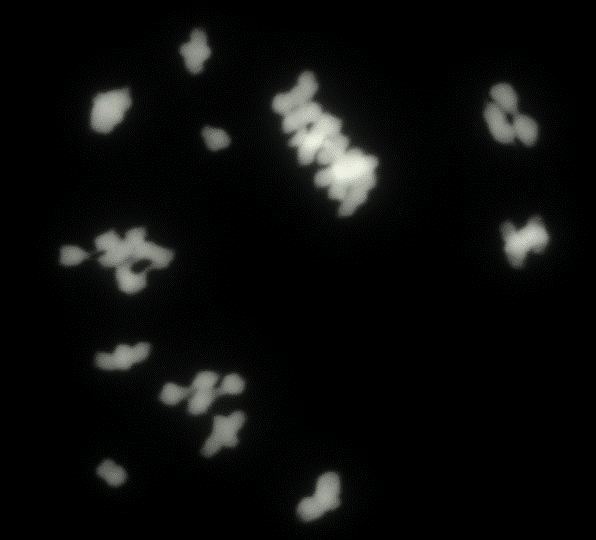


1

1

2

a


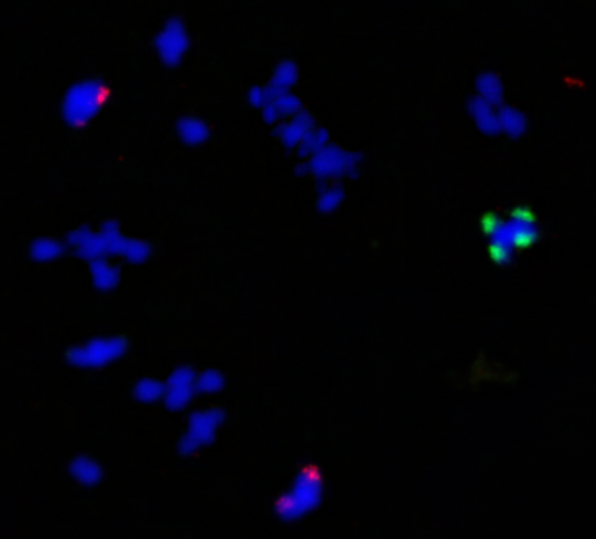


IV

II

II

b


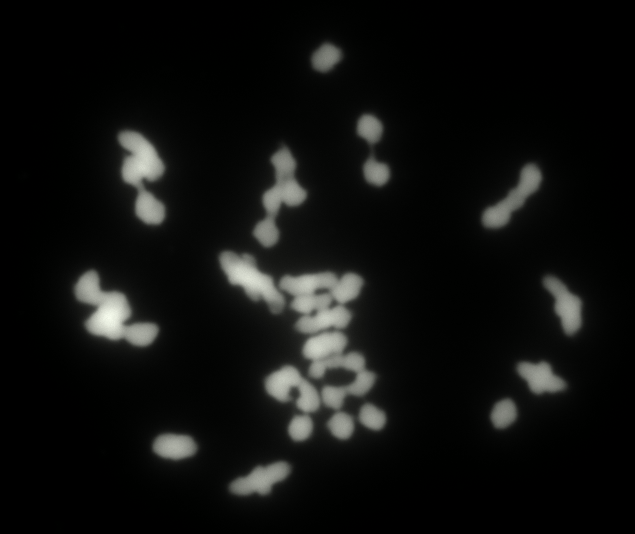


1

2

c


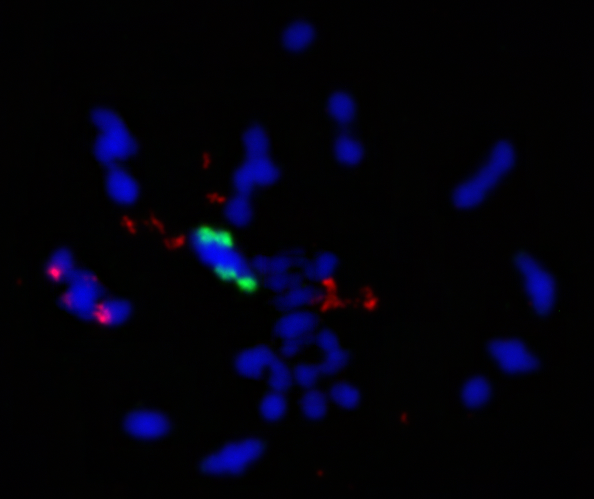


IV

IV

d

FISH signals were detected in metaphase I cells using 5S rDNA probe (red) for chromosome 1 and 45S rDNA probe (green) for chromosome 2. In **a)**-**b)**, chromosome 1 shows two bivalents (II, 1 rod, 1 ring), while chromosome 2 is a ring quadrivalent (IV). In **c)**-**d)**, chromosome 1 is a chain quadrivalent while chromosome 2 is a ring quadrivalent. Chromosomes have been stained with DAPI (blue). Scale bars = 10µm.

**Fig. S3** Chiasma counting for all 12 potato chromosomes in tetraploid varieties Maris Peer and Sante


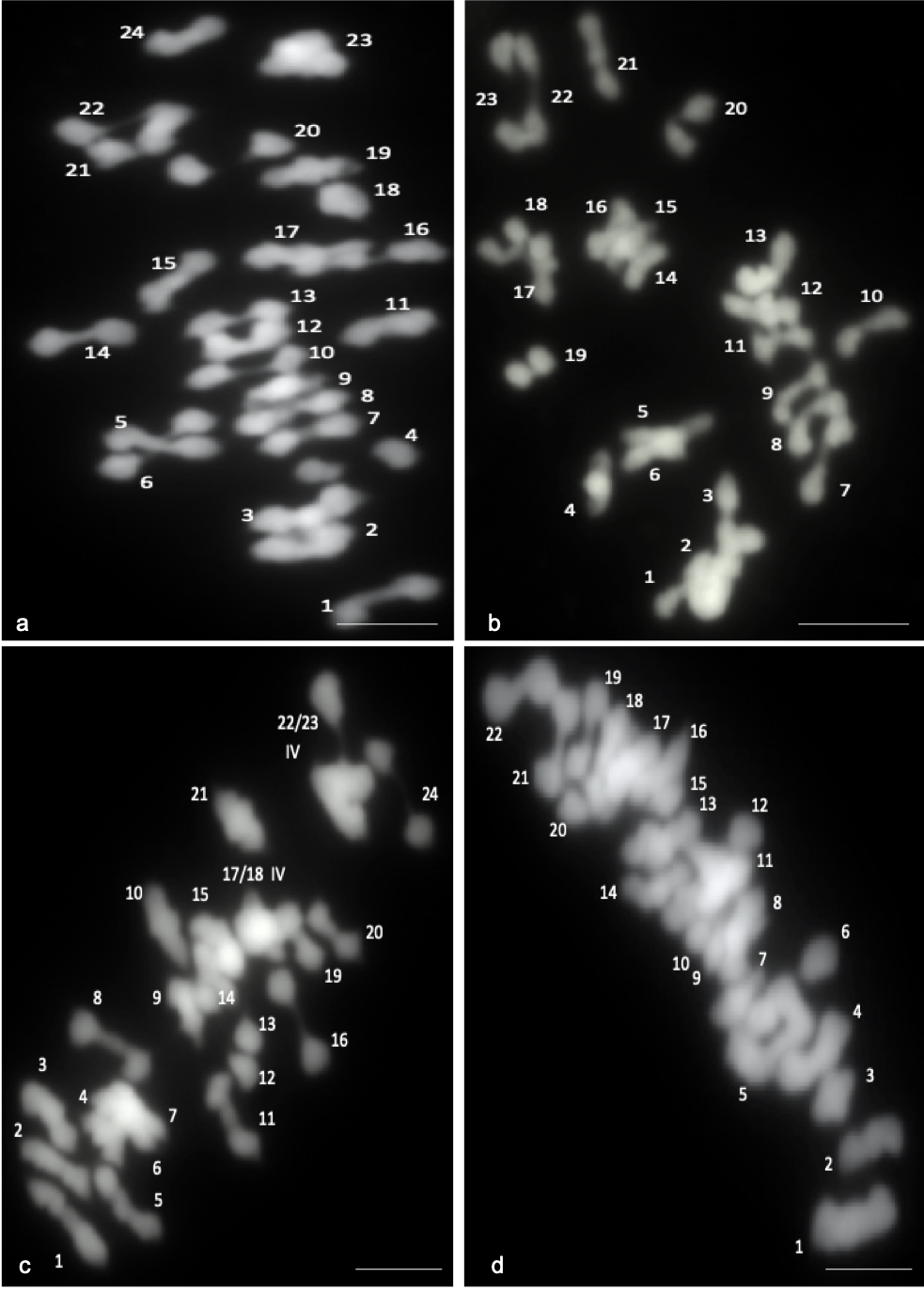


In a minority of tetraploid cells, all 48 chromosomes can be observed as 24 units pairs labelled with roman numerals, consisting of either 24 bivalents or a combination of bivalents and multivalents in Maris Peer (**a**) and Sante (**c**). For other cells, the 24 unit pairs cannot be observed, often due to a more crowded arrangement of chromosomes, and so a total chiasma count per cell is not made, as shown in Maris Peer (**b**) and Sante (**d**). Scale bars = 10µm.

**Fig. S4** BLAST comparison between *Arabidopsis thaliana* (subject) and *Solanum tuberosum* (query) protein sequences for meiotic axis protein ASY1 (**a**) and synaptonemal complex protein ZYP1 (**b**). Percent identity indicates the exact amino acid match and similarity indicates a substitution in amino acid with similar physicochemical properties (Madden, 2002).


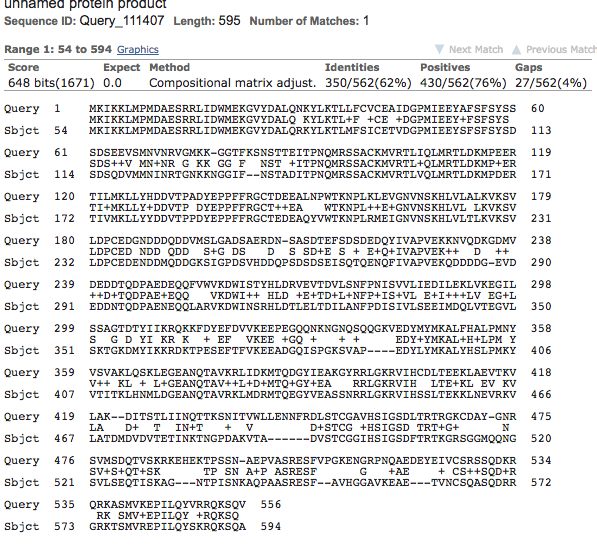
**a)**

**b)**


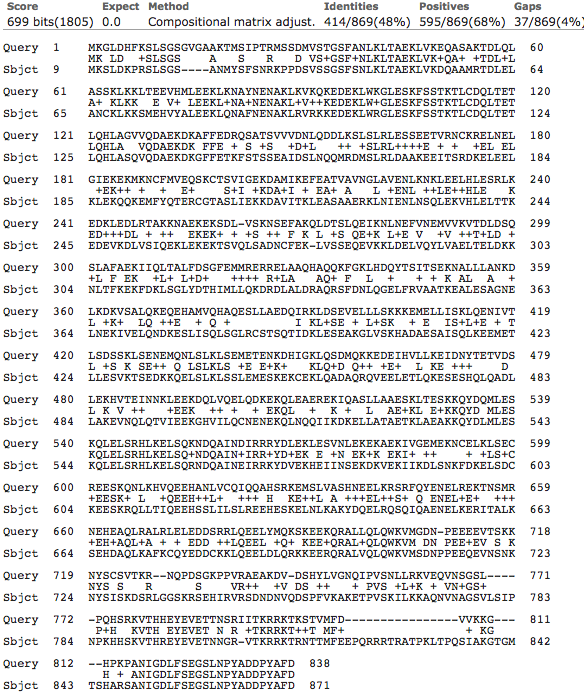


**Fig. S5** Greyscale versions of the FISH figures 2, 6 and 7

1. Figure 2

**
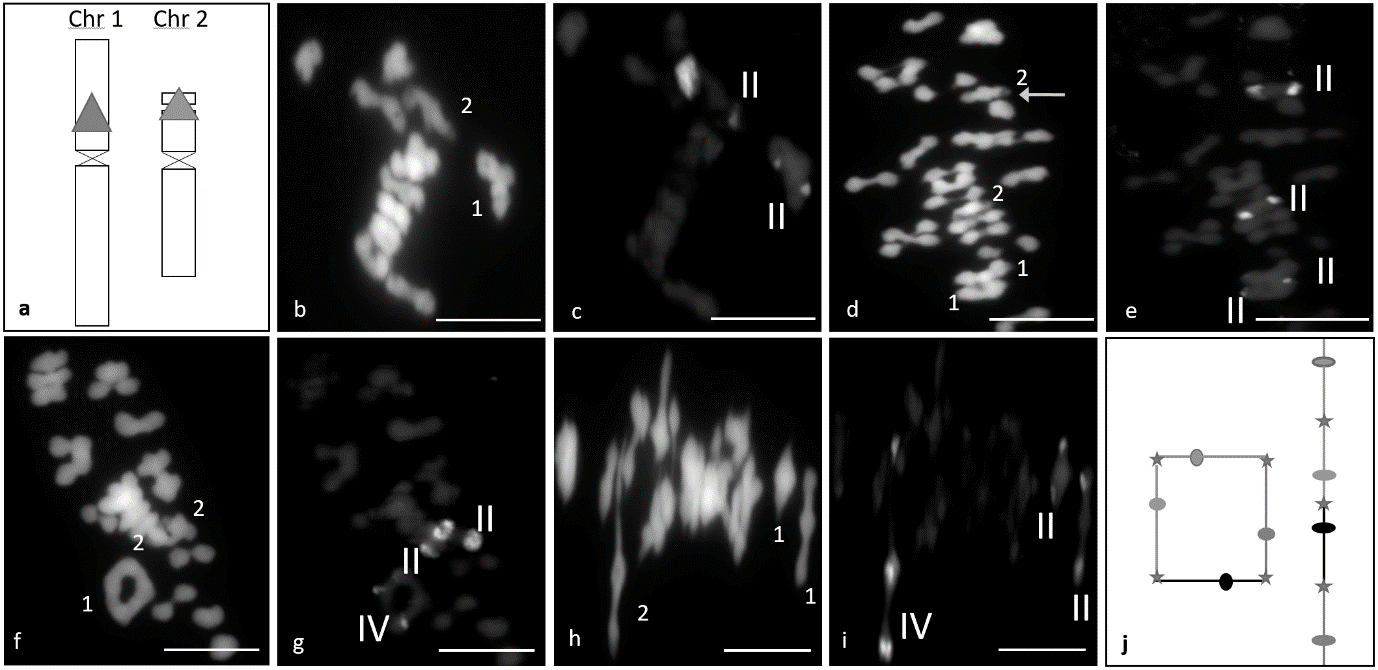
**

1. Figure 6

**
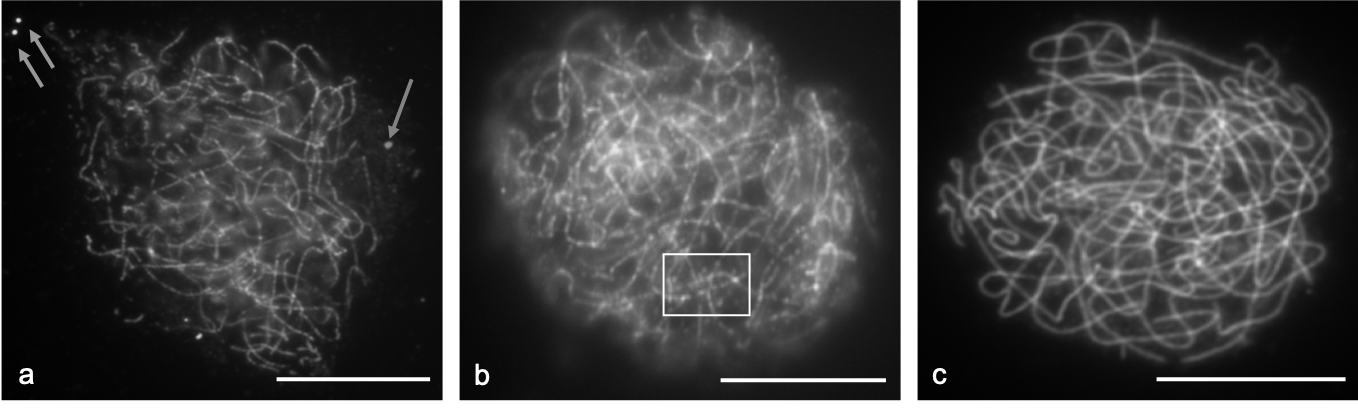
**

1. Figure 7


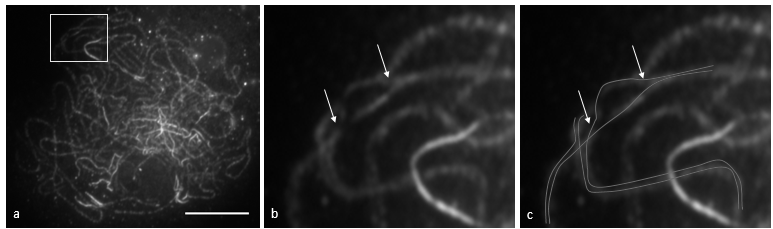


**Supplementary Tables**

**Table S1** Characteristics of tetraploid potato germplasm used in the current study

| Kinship Group (K) | Variety | Market class/Purpose | French Fry | Crisp | After Cooking Darkening | Taste | Dry Matter | Starch | Year of release | Maturity | Origin |
| --- | --- | --- | --- | --- | --- | --- | --- | --- | --- | --- | --- |
| 3**^a^** | Sante | Multi-purpose/Floury | Poor/Mod/Good | Poor/Mod/Good | Trace/Little | Mod/Good | Med/High | Med/High | 1983 | Early/Int/Late | Netherlands |
| 7**^b^** | Maris Peer | - | Poor | Poor | None | - | Low/Med | Medium | 1962 | Early | UK |
| 10**^c^** | Cara | Multi/Floury/Salad | Poor | Poor | None/Trace | Good | Low/Med | Low | 1973 | Late/V Late | Ireland |

Modified from Sharma et al. 2018. Kinship groups were identified by Sharma et al. (2018) from a genomic relationship matrix constructed using Gower’s similarity metric.

**a** Kinship group 3 consists mainly of Dutch, UK and German cultivars (kinship groups 3-5).

**b** Kinship group 7 consists mainly of older UK cultivars (kinship groups 7-8).

**c** Kinship group 10 consists mainly of more modern UK and Irish cultivars (kinship groups 9-10).

**Table S2** Anther size and meiotic stages in diploid and tetraploid S. tuberosum

| **Anther size**  **(mm)** | **Diploid**  **(meiotic stage)** | **Tetraploid**  **(meiotic stage)** |
| --- | --- | --- |
| 1.5 | G2 and leptotene | G2 |
| 1.6 | Leptotene-zygotene | Leptotene-zygotene |
| 1.65-1.7 | Leptotene-pachytene | Leptotene-zygotene |
| 1.7-1.85 | Leptotene-pachytene | Zygotene-pachytene |
| 1.9-2.0 | Diakinesis-tetrad | Diakinesis-metaphase I |
| 2.0-2.1 | Metaphase II-tetrad | Metaphase I-tetrad |

| Variety (4x) | Chr. | χ^2^ _df=1_ | p-value | *n* |
| --- | --- | --- | --- | --- |
| Cara  Cara | 1 | 3.83 | 0.05 | 27 |
|  | 2 | 19.32 | <0.0001 | 27 |
| Sante  Sante | 1 | 76.27 | <0.0001 | 71 |
|  | 2 | 85.28 | <0.0001 | 71 |
| Maris Peer  Maris Peer | 1 | 82.85 | <0.0001 | 305 |
|  | 2 | 181.00 | <0.0001 | 305 |

**Table S3** Chi square goodness of fit to the random end model

The random end model is defined by a predicted 2:1 ratio of multivalents to bivalents, where multivalents include quadrivalents and trivalents (Sybenga 1975). *n* is the number of pollen mother cells analysed.

**Table S4** *Post hoc* Dunn test p values (after Kruskall-Wallis test) for comparison of variation in chiasma frequency among varieties

1. *overall* chiasma frequency

|  |  | Cara (4x) (n=27) | | Maris Peer (4x) (n=305) | | Sante (4x) (n=71) | |
| --- | --- | --- | --- | --- | --- | --- | --- |
|  |  | Chr. 1 | Chr. 2 | Chr. 1 | Chr. 2 | Chr. 1 | Chr. 2 |
| Maris  Peer (4x)  (n=305) | Short  Long  Total | 1.00  1.00  1.00 | 1.00  1.00  1.00 | - | - | - | - |
| Sante  (4x)  (n=71) | Short  Long  Total | 1.3x10^-2^ *  1.00  0.16 | 0.14  1.00  0.48 | 2.7x10^-3^ **  1.00  0.19 | 3.0x10^-2^ *  1.00  0.56 | - | - |
| Scapa  (2x)  (n=236) | Short  Long  Total | 3.3x10^-4^ ***  2.5x10^-20^ ***  5.8x10^-17^ *** | 4.5x10^-2^ *  2.1x10^-22^ ***  7.5x10^-19^ *** | 6.3x10^-11^ ***  6.9x10^-98^ ***  2.0x10^-68^ *** | 2.6x10^-5^ ***  1.1x10^-102^***  1.4x10^-81^ *** | 1.00  1.8x10^-45^ ***  3.1x10^-19^ *** | 1.00  1.3x10^-41^ ***  7.7x10^-26^ *** |

Bonferroni corrected p-values are given for the comparison of *overall* chiasma frequency, including all cells in tetraploids regardless of chromosome pairing configuration. The Kruskal-Wallis test results for chromosome 1 (short arm: K-W χ^2^_df=3_ = 55.65, p < 5.0 x 10^-12^; long arm: K-W χ^2^_df=3_ = 511.32, p < 2.2 x 10^-16^; total: K-W χ^2^_df=3_ = 336.47, p < 1.3 x 10^-72^) and for chromosome 2 (short arm: K-W χ^2^_df=3_ = 26.58, p < 7.2 x 10^-6^; long arm: K-W χ^2^_df=3_ = 525.17, p < 2.2 x 10^-16^; total: K-W χ^2^_df=3_ = 403.96, p < 3.1 x 10^-87^). Significance is indicated by * p < 0.05; ** p < 0.01; *** p < 0.001. *n* is the number of pollen mother cells analysed.

1. chiasma frequency *per chromosome*

|  |  | Cara (4x) (n=27) | | Maris Peer (4x) (n=305) | | Sante (4x) (n=71) | |
| --- | --- | --- | --- | --- | --- | --- | --- |
|  |  | Chr. 1 | Chr. 2 | Chr. 1 | Chr. 2 | Chr. 1 | Chr. 2 |
| Maris  Peer (4x)  (n=305) | Short  Long  Total | 1.00  1.00  1.00 | 1.00  0.85  1.00 | - | - | - | - |
| Sante  (4x)  (n=71) | Short  Long  Total | 0.06  1.00  6.4x10^-2^ * | 0.20  0.66  2.2x10^-2^ * | 2.2x10^-2^ *  0.83  0.08 | 0.05 *  1.00  2.1x10^-2^ * | - | - |
| Scapa  (2x)  (n=236) | Short  Long  Total | 1.00  1.00  1.00 | 0.30  1.00  0.09 | 1.00  0.24  1.00 | 1.4x10^-2^ *  0.37  0.07 | 8.0x10^-3^ **  1.00  3.2x10^-2^ * | 1.00  0.56  1.00 |

Bonferroni corrected p-values are given for the comparison of chiasma frequency *per chromosome*, including all cells in tetraploids regardless of chromosome pairing configuration. The chiasma frequency per chromosome is obtained by dividing the *overall* chiasma frequency by four in tetraploids, or by 2 in the diploid. The Kruskal-Wallis test results for chromosome 1 (short arm K-W χ^2^_df=3_ = 11.94, p < 7.6 x 10^-3^; long arm: K-W χ^2^_df=3_ = 5.22, p < 0.16; total: K-W χ^2^_df=3_ = 9.86, p < 2.0 x 10^-2^) and for chromosome 2 (short arm: K-W χ^2^_df=3_ = 14.58, p < 2.2 x 10^-3^; long arm: K-W χ^2^_df=3_ = 6.14, p < 0.11; total: K-W χ^2^_df=3_ < 15.81, p < 1.2 x 10^-3^). Significance is indicated by * p < 0.05; ** p < 0.01; *** p < 0.001. *n* is the number of pollen mother cells analysed.

1. *per bivalent* chiasma frequency

|  |  | Cara (4x)  (n_b1_=14, n_b2_=20) | | Maris Peer (4x)  (n_b1_=178, n_b2_=208) | | Sante (4x)  (n_b1_=59, n_b2_=60) | |
| --- | --- | --- | --- | --- | --- | --- | --- |
|  |  | Chr. 1 | Chr. 2 | Chr. 1 | Chr. 2 | Chr. 1 | Chr. 2 |
| Maris  Peer (4x) | Short  Long  Total | 1.00  1.00  1.00 | 0.90  1.00  0.13 | - | - | - | - |
| Sante  (4x) | Short  Long  Total | 1.00  1.00  1.00 | 0.65  1.00  0.40 | 1.00  1.00  1.00 | 1.00  1.00  1.00 | - | - |
| Scapa  (2x)  (n=236) | Short  Long  Total | 1.00  1.00  1.00 | 1.00  1.00  1.00 | 2.7x10^-3^ **  1.00  5.3x10^-3^ ** | 1.9x10^-3^ **  1.00  5.1x10^-5^ *** | 9.4x10^-4^ ***  1.00  3.9x10^-3^ ** | 2.1x10^-2^ *  1.00  0.08 |

Bonferroni corrected p-values are given for the comparison of *per bivalent* chiasma frequency. Only tetraploid cells showing two bivalents for chromosome 1 (n_b1_) or chromosome 2 (n_b2_) are included. The Kruskal-Wallis test results for chromosome 1 (short arm K-W χ^2^_df=3_ = 20.63, p < 1.3x10^-4^; long arm: K-W χ^2^_df=3_ = 2.08, p < 0.55; total: K-W χ^2^_df=3_ = 17.59, p < 5.34 x 10^-3^) and for chromosome 2 (short arm: K-W χ^2^_df=3_ = 17.28, p < 6.4 x 10^-4^; long arm: K-W χ^2^_df=3_ = 4.95, p < 0.18; total: K-W χ^2^_df=3_ = 23.31, p < 3.48 x 10^-5^). Significance is indicated by * p < 0.05; ** p < 0.01; *** p < 0.001.

**Table S5** Mean chiasma frequency for all 12 chromosomes in three *S. tuberosum* varieties

1. Mean chiasma frequency per cell or per chromosome

| Variety  (Number of cells) | Mean chiasma frequency | |
| --- | --- | --- |
|  | per cell | per chromosome |
| Sante, 4x (n=30) | 27.4 | 6.9 |
| Maris Peer, 4x (n=10) | 30.3 | 7.6 |
| Scapa, 2x (n=126) | 15.2 | 7.6 |

The mean chiasma frequency is given for all pollen mother cells in each variety (*n*) in which all 48 (tetraploids) or 24 (diploids) chromosomes could be clearly observed. In tetraploid variety Cara there were no such cells (*n* = 0). The mean chiasma frequency per cell includes all cells in tetraploids regardless of chromosome configuration. The chiasma frequency per chromosome is obtained by dividing the overall frequency per cell by four in tetraploids, or by 2 in the diploid.

1. *Post hoc* Dunn test p values (after Kruskall-Wallis test) for comparison of variation in chiasma frequency

|  |  | Sante (4x) (n=30) | Maris Peer (4x) (n=10) |
| --- | --- | --- | --- |
| Sante (4x) (n=30) | per cell  per chrom. | -  - | -  - |
| Maris Peer (4x)(n=10) | per cell  per chrom. | 1.00  0.04 * | -  - |
| Scapa (2x) (n=126) | per cell  per chrom. | 3.2x10^-16^ ***  1.5x10^-5^ *** | 2.2x10^-8^ ***  1.00 |

Bonferroni corrected p-values are given for the comparison of overall chiasma frequency per cell (K-W χ^2^_df=2_ = 92.19, p < 2.2 x 10^-16^) or per chromosome (K-W χ^2^_df=2_ = 21.01, p < 2.7 x 10^-5^). Significance is indicated by * p < 0.05; ** p < 0.01; *** p < 0.001. *n* is the number of pollen mother cells analysed.

**Supplementary References**

Madden T The BLAST Sequence Analysis Tool. 2002 Oct 9 [Updated 2003 Aug 13]. In: McEntyre J, Ostell J, editors. The NCBI Handbook [Internet]. Bethesda (MD): National Center for Biotechnology Information (US); 2002-. Chapter 16. Available from: <https://www.ncbi.nlm.nih.gov/books/NBK21097/>

Sharma SK, MacKenzie K, McLean K, Dale F, Daniels S, Bryan GJ (2018) Linkage disequilbrium and evaluation of genome-wide association mapping models in tetraploid potato. G3 (Bethesda) 8:3185-3202

Sybenga J (1975) The Analysis of Chromosome Pairing, in Meiotic Configurations: A Source of Information for Estimating Genetic Parameters. Berlin, Heidelberg: Springer, pp 134–199
